# The Value of Sanatorium Treatment

**Published:** 1909-04-24

**Authors:** Arthur Latham

**Affiliations:** Physician to St. George's Hospital


### HOSPITAL CLINICS.

### THE VALUE OF SANATORIUM TREATMENT.

By ARTHUR LATHAM, M.D., F.R.C.P., Physician to St. George's Hospital.

A Paper read before the International Congress on Tuberculosis at Washington, 1908.

SOME years ago I expressed the opinion that sanatoriums are essential to the successful treatment of pulmonary tuberculosis on a large scale, and that these institutions afford invaluable aid to any campaign directed towards the eradication of the disease, provided they are used intelligently as an important link in a properly co-ordinated system of attack. After several years of increased experience I see no reason to withdraw this opinion: on the contrary, I am more convinced than ever of its truth.

The fact that this opinion does not meet with universal acceptance is due to several causes. In the first place, the majority of sanatoriums are inefficient. Many of these institutions are mere hotels run for profit, and do not provide the discipline and constant medical supervision which are so essential to success. The value of a sanatorium depends on the man at its head, and no sanatorium in my experience is efficient when the head of it resides at a distance, or has his powers and discretion fettered by either a lay or a medical committee. Much of the criticism of sanatorium treatment is based upon the erroneous deduction that because certain sanatoriums fail to give good results, therefore sanatorium treatment is a failure.

The second reason why the value of sanatorium treatment does not meet with universal acceptance is the fact that sanatoriums have been regarded in some quarters as sufficient in themselves, and treatment therein as a certain cure for every case of consumption. No level-headed man would ever have regarded sanatorium treatment in this light. It is clear that a sanatorium is a link in a comparatively long chain, and that unless it forms a part of a carefully thought out system, embracing amongst other things dispensaries, hospitals, homes for the dying and advanced cases, careful disinfection, and after-care, together with assistance to the breadwinner's family during his absence, it cannot bring about the eradication of tuberculosis. Those who have taught the public to regard sanatorium treatment as a specific cure for all stages and forms of pulmonary tuberculosis will be, and, in fact, have already been, discredited; but—and this is of more importance—sanatorium treatment has to a certain extent fallen into disrepute amongst the less intelligent members of the public owing to this teaching. Sanatorium treatment does not cure all forms of pulmonary tuberculosis, and no form of treatment ever will cure all cases of advanced and long-standing disease. Is it fair to condemn sanatorium treatment because it cannot accomplish the impossible? Again, if a working man in an early stage of consumption is sent to an efficient sanatorium, there is every prospect of the disease being arrested; but if he is allowed to return to precisely those conditions under which he contracted the disease—too hard work, bad air, insufficient food—the disease will almost certainly return. Is it fair when this happens to say that sanatorium treatment is of no avail?

The third reason why sanatorium treatment does not meet with universal acceptance is that the erection and the maintenance of sanatoriums have cost too much. The erection of costly palaces for the treatment of consumption in the past is a standing monument to the possibilities of human folly. Fortunately this wasteful expenditure of money is now a thing of the past. Mr. A. William West (architect) has shown that it is possible to erect an efficient and comfortable sanatorium for 200 persons at a total cost, for erection and equipment (apart from the cost of land), of £100 a bed. It may be mentioned, in order to show the durability of his construction, that in the Benenden sanatorium for members of the working classes the floors and doors are made of teak. The building of a sanatorium at a reasonable price has been accomplished by Mr. West by means of a special steel girder construction which does away with the necessity for expensive foundations, and makes it possible for the larger part of the labour to be carried out by unskilled workmen. Again, Dr. Paterson has shown that it is possible to conduct the Brompton Hospital sanatorium at Frimley at a cost of 25s. per week per patient for maintenance and administration. This has been accomplished largely by his system of graduated labour, whereby the greater part of the labour necessary for the upkeep and cleaning of the sanatorium is carried out by the patients themselves, with marked benefit to their health.

The fourth reason why the value of sanatorium treatment does not meet with universal acceptance is that it is held by many that it is impossible to provide the necessary funds to pay for the treatment of the majority of sufferers. The German insurance system has shown the inadequacy of this argument. Again, Mr. C. H. Garland has organised a fund amongst the employees of the British Post Office, which meets the cost of sanatorium treatment for all such employees who contract consumption by an individual contribution of ½d. a week, which is deducted at headquarters from their wages. It is interesting to note that nearly 40,000 employees are contributing to this scheme. There is no reason why a scheme of this kind should not be in existence amongst all skilled workmen.

The final objection is the difficulty in arranging for the maintenance of the family when the breadwinner is away, and in finding suitable work for him on his return. The German Government have solved this problem and other countries can do the same. The employees of the British Post Office who contribute to the scheme outlined above receive full pay from the Government for six months when away at a sanatorium, for the provision of their wives and families. At the Brompton Hospital the Lady Almoner supplies the necessary link between the patient and his employer, the clergy, and the various charitable bodies, and in this way the needs of his wife and family are met without much difficulty. Dr. Paterson's system of graduated labour has shown that it is possible to treat a man in such a way that the muscles used in his ordinary work are kept trained, and the patient is enabled to resume his previous occupation immediately after his discharge.

The best proof of the value of sanatorium treatment is obtained from a study of the results of treatment before and after the adoption of sanatorium methods. Dr. J. E. Pollock published in 1865 the results of treatment in 3,566 cases of consumption, in all stages, which had been observed by himself. The average duration of life of all these cases, from the first symptom of the disease to the fatal termination, was two and a half years. Dr. Pollock further found that no less than 83 per cent. of the patients whom he saw in the early stage of the disease died within five years of their first symptom. Sanatorium treatment has completely altered this grim picture. Thus in 1905 no less than 57 per cent. of the patients who were treated in 1900 in the Prussian and Hessian State Railway sanatoriums were capable of full work. We see, then, that before the advent of sanatorium treatment 83 per cent. of patients in an early stage were dead at the end of five years, and that after its advent 57 per cent. remained in full enjoyment of their working capacity at the end of five years.

More recent statistics tell the same tale, and also show that by combining sanatorium with tuberculin treatment still better results can be obtained, whilst the percentage of relapses is still further diminished. Thus Dr. Ritter, of the Hamburg Heilstätte Edmundsthal, has found that patients who had been treated by sanatorium methods alone showed the following capacity for full work three years after their discharge from the sanatorium, namely:—

| | | |
|---|---|---|
| Patients treated in first stage of disease | ... | 72% at work |
| " second " " | ... | 57 " |
| " third " " | ... | 22 " |

Patients who had been treated by sanatorium methods, together with tuberculin, showed the following capacity for full work three years after their discharge from the sanatorium:—

| | | |
|---|---|---|
| Patients treated in first stage of disease | ... | 95% at work |
| " second " " | ... | 82 " |
| " third " " | ... | 50 " |

No criticism can ever overcome the force of these statistics, but the value of sanatorium treatment is not confined to the immediate effects produced on the patients. One of its greatest features is its educational value. Sanatorium treatment has done more than anything else to awaken the public to the necessity of hygienic conditions of life. This is partly due to the notice taken by the public press of the movement, and partly to the fact that every patient who leaves a sanatorium acts as a missionary and preaches the value of hygienic living and the necessity of preventing the spread of infection.

We have known for many years that the continuous supply of fresh air, good hygienic conditions, and a nutritious diet increase the resisting capacity of the tissues. We have also known for many years that the success of sanatorium treatment depends largely on the regulation of the amount of rest and exercise prescribed for each patient. We did not understand exactly how exercise does good, or why in many instances it does harm. Dr. A. C. Inman, by his study of the opsonic index of the patients performing the different grades of labour prescribed for them by Dr. Paterson at the Frimley Sanatorium has given us an explanation which is at once reasonable and scientific. This explanation shows clearly that the reaction of a patient to the disease depends very largely upon the question whether the amount of tuberculin absorbed from the diseased focus is beyond the immunising capacity of the body or not. Excessive absorption—*i.e.* excessive auto-inoculation—means steady progress of the disease, accompanied with fever. The amount of this absorption or auto-inoculation depends very much on the amount of movement permitted. If the amount of auto-inoculation is sufficient and not too great the body tissues are stimulated and opsonins and other defensive bodies are manufactured. After a time the tissues fail to respond to this stimulus, and consequently a larger stimulus is required. This larger stimulus is produced by increasing the exercise, which increases the absorption of the tuberculous products. It is in this fact that we have the secret of the success of those sanatoriums in which the patients do better than the patients of other sanatoriums simply because they are *gradually* educated to walk as far as 20 miles a day, and are not considered to have reached a satisfactory condition when only five or six miles a day have been accomplished. Here also we have the explanation of the success of Dr. Paterson's scheme of graduated labour. Too large a stimulus—*i.e.* excessive auto-inoculation—renders the body tissues incapable of response. Such a stimulus is due to excessive movement, and can only be met by rest. The decision as to the amount of exercise or movement permitted to an individual patient thus requires great judgment and discretion. Constant medical supervision alone can decide this question correctly. This constant attention to every detail, and especially to the amount of movement permitted, is the key to success. It is clearly possible to give a patient sanatorium treatment without sending him to a sanatorium. Most patients, however, are more likely to carry out the treatment in every detail if they are in an institution entirely given up to this purpose, for it is hard to make them understand the importance of continuously carrying out all the directions given unless they are first sent to school to learn how to get well. At the same time, patients of strong character with the necessary home facilities who intend to get well can benefit almost if not quite as much by treatment under close medical supervision at their own homes.

